# “My Tears Have Been My Food, Both Day and Night”: Integrating Theology and Psychology on the Nature of Grief

**DOI:** 10.3390/rel16111353

**Published:** 2025-10-27

**Authors:** Daniel Lee Hill, Sierra Wickline, Angie S. LeRoy

**Affiliations:** 1 George W. Truett Seminary, Baylor University, Baylor, TX 76798, USA; 2 Department of Psychological & Brain Sciences, University of North Florida, 1 UNF Drive, Jacksonville, FL 32224, USA;

**Keywords:** grief, theodicy, Augustine, suffering, theological description, selfhood, bereavement

## Abstract

Recent work on theological reflection on the phenomenon of human grief has called for an apophatic posture, one that resists speaking positively about what grief is, what grief does, or where it is situated with respect to the economy of God’s works. Grief, on this account, is viewed as inaccessible to theological inquiry and illogical in nature. This essay references recent work in psychological science to offer “theological fragments” on the nature of grief with the respect to the generational self’s journey in the “time of weeping.” In so doing, it argues that an integration of theological and psychological science provides a framework for describing grief in a way that resists the temptation to ascribe positive meaning or value to it in the reconciling work of God.

## 1. Introduction

Theological engagement with the problems of evil and suffering in recent years seems to have become increasingly disenchanted with the promise of theodicy. So, while someone like Julian of Norwich or Gregory of Nyssa might be able to positively assess suffering’s value, such sentiments increasingly fall on numb and skeptical ears in the twentieth and twenty-first centuries (Gregory of Nyssa 2005; Julian of Norwich 2015). Part of this may be indicative of the fact that the existential shape of theodicy has taken in the post-Enlightenment era, as Mara van der Lugt has noted (van der Lugt 2021, pp. 20–21). Nevertheless, theologians seem increasingly dissatisfied with the prospects of theodicy and, while some still offer arguments and defenses for God’s goodness in light of the problems of evil and suffering,^1^ many have identified a boundary where theological speech must recognize its limit.^2^

What is true of the problems of evil and suffering more broadly, might be applied to the grief human creatures feel in response to the aforementioned sufferings and evils. That is, attempts to analyze grief with the tools of theological reason might be viewed as guilty of a kind of epistemic overreach. And insofar as theological inquiry seeks to provide a rational account of God and his ways usward, it is limited in its engagement with this phenomenon. But is this the only kind of theological description on offer? Might theological resources be brought to bear on the issue of grief and suffering, without positing any positive valuation or meaning in light of God’s ultimate triumph over the forces of death? In this essay, we will argue that while the nature of grief renders it a complex and recalcitrant phenomenon that is resistant to exhaustive theological normative analysis, theological engagement and integration with psychological science provides resources for theologically describing grief “in fragments,” that is, developing salient yet incomprehensive accounts and models of the nature of grief as it pertains to the disintegration and reintegration of the self in the “time of grief.”^3^ In other words, grief may elide thick and exhaustive theological description, but an integration with the insights of psychological science can provide resources for thinking about the different expressions, components, and processes of the grieving person. This essay will unfold in four parts. In the first part, we will raise the problem grief poses to theological analysis, as articulated in the work of Karen Kilby and William Abraham. Both Kilby and Abraham agree that grief and suffering are resistant to theological analysis precisely because of its inaccessibility from a third-person perspective. An inaccessibility that renders it fundamentally unintelligible to outside observers. The next section turns to engage the literature of psychological science on the issue of grief, focusing specifically on the nature of bereavement, the manner in which it is observable in human behavior and physiology, and the insights that attachment theory offers for analyzing why human creatures grieve and find themselves disordered by grief. The paper’s final section seeks to build on this psychological data to develop two “fragments” or “vectors” for a theological description of grief.

## 2. The Intractable Problem(s) of Grief

Galen Strawson, in his recent book *Things That Bother Me*, posits that the phenomenon of death and loss should not trouble us, as our “future life and experience … don’t belong to [us] in such a way that they’re something that can be taken away” (Strawson 2018, p. 72). Ostensibly, the same would hold true for the death and loss of those we love and any experiences of grief pertaining thereto. We cannot, on Strawson’s account, grieve a lost future with those we love because that future does not belong to us in any robust sense (Strawson 2018, p. 75). Indeed, Parfitt goes so far as to suggest that such rational reflection on identity and time, and presumably on the nature of the future, can then mitigate the intensity of grief (Parfit 1986, pp. 305–19, 451).

Yet such a disposition toward the ameliorative potential of rational reflection on loss is rarely shared with their theological colleagues. Indeed, in recent years, Karen Kilby has written extensively on the limits of theological science, especially with respect to the problems pertaining to the existence of sin, evil, and suffering as well as theological attempts to make sense of these phenomena (Kilby 2020a, chaps. 2, 6 and 7). For Kilby, these limitations of theological reflection not only apply to the doctrine of, say, the nature of the processions of the Trinitarian persons, but also to the problems of sin, evil, and the attendant marks they leave on the world in the form of suffering and grief. Critical of attempts to discover theological grounds for suffering or evil in Godself, Kilby writes, “it is the nature of Christian theology to make affirmations, or patterns of affirmations—about the goodness, faithfulness and creative power of God on the one hand, and the brokenness of creation on the other—that it cannot co-ordinate or make sense of. There are points, then, at which systematic theology (if there is such a thing) ought to be, if not systematically incoherent, then at least systematically dissonant” (Kilby 2020a, p. 81). Suffering and evil, in general, and grief, specifically, are elements of creaturely existence that cannot be made sense of, that resist any attempt to demonstrate how they all hang together within the plan and purposes of God.^4^

As it relates to the question of suffering and its meaning, Kilby posits that our perspective and relationship to that suffering is vital. She distinguishes between “the place of speech and thought *within* suffering” as opposed to “speech and thought *towards* suffering” (Kilby 2020b, pp. 97–98). Thus, we must distinguish speech about suffering in a first-, second-, or third-person perspective. According to Kilby, theological discourse operates primarily in this third category, describing the human creature and attempting to situate her with respect to God and God’s ways usward, all of which functions in a third-person perspective (e.g., “her,” “it,” “them,” “God,” “The Holy Spirit,” etc.). This is a problem, then, for the theologian who seeks to reflect upon suffering and its attendant forms, of which grief and mourning would be enumerated, because she must do so in a way that almost necessarily violates the experience of suffering itself and violates the limit of what the theologian can and ought to do (Kilby 2017, p. 288). Kilby writes, “it is clear that it makes a vital difference, in any talk about suffering, whether I am talking about my suffering, or yours, or theirs—whether I am in a first- or second- or third-person relation to suffering… There are certain things that can be said in relation to *my* suffering … that usually can*not* be said in relation to yours or to theirs” (Kilby 2020c, p. 169). For Kilby, there is a personal boundary or limit with respect to the issue of suffering, one which ought to restrain theological discourse about it (Kilby 2017, p. 288). So, Kilby writes, “the process of finding some way to be at least partly reconciled to suffering, of fitting it into a larger pattern of value, of discerning grace and growth met within suffering, is for the most part not one we can properly undertake in the second or third person” (Kilby 2020b, p. 98).

Instead, Kilby calls for a kind of apophatic restraint if not silence in the face of such suffering and grief and a resistance to weave them into greater narratives of meaning and divine purpose (Kilby 2020c, p. 174). While a particular sufferer may feel that they have glimpses or intimations of God’s presence and work in the midst of their suffering (first-personally), Kilby avers that for an outside observer to suggest as much involves a kind of moral insensitivity and moral failure, it involves “approaching the suffering of others in a way [we] have no right to do” (Kilby 2017, p. 289). Recognizing the pull that theologians feel to say something “in the face of the suffering of another, especially when one has no cure, no fix,” Kilby advocates for the cultivation of “a certain ascesis, a certain restraint, the capacity not to find meaning where it does not exist, or where we have no right to it” (Kilby 2020c, pp. 172, 174). Insofar as grief is a form suffering, we must attend the limits of theological speech to engage and describe the phenomenon, both presently and its salience to the world without end. Indeed, for Kilby there is a kind of “apophatic pressure” on the eschatological imagination *en toto*, not just with respect to the problems of evil, grief, and suffering. “Our capacity to speak, to speculate, to theorize, is limited, caught up short, called into question, *both* by the mystery of God *and* by the mystery of suffering” (Kilby 2017, p. 291).

William Abraham, reflecting on the tragic and unexpected nature of his son’s death, concretizes some of Kilby’s reflection on suffering and tethers them specifically to his own experience of grief. Familiar with the literature on grief and mourning, Abraham notes that it “uses all sorts of images to capture the suffering that occurs on occasions like this. A favorite one is the loss of a limb. Mine is equally simple: to lose Timothy was to fall precipitously into a deep black hole. It was a hole of darkness, numbness, despair, and waves of excruciating pain” (Abraham 2017, p. 4). Abraham received an outpouring of support and answered prayers in the moments surrounding his son’s death. Yet this did little to attenuate the pain he felt in the wake of the loss of his son. Like Kilby, Abraham’s reflections on his experience with grief direct him to question the salience of theodicy with respect to sin, evil, and grief. He writes, “I know all the standard moves in theodicy and even endorse a robust and integrated set of those moves; I do not sense that these are somehow irrelevant to my situation; what I find is that they are incapable of doing any work in my life. What is at issue is that I found every philosophical and theological move utterly empty in wrestling with the problem of the loss of my son” (Abraham 2017, p. 11).

The intractable nature of Abraham’s experience with grief leads him to infer that theodicy, that is, the attempt to offer rational descriptions and analysis of pain or suffering from a philosophical or theological vantage point, is inadequate and irrelevant to grief because it occurs in the wrong theological register. He writes, “In this situation any philosophical or theological discourse from a third-person perspective fails. To be told that God permits it for his good purposes, or that Timothy is now in the hands of his Savior, or that good things will come out of this suffering, or that we walk by faith and not by sight, has no purchase on us intellectually” (Abraham 2017, p. 15). According to Abraham, this is because the nature of grief or suffering leads to a kind of cognitive or intellectual disruption (Abraham 2017, p. 16). In the wake of tragic loss, Abraham posits that “our cognitive capacities, powers, and skills have lost their moorings … we are operating in a radically abnormal epistemic environment” (Abraham 2017, p. 17). In fact, due the very nature of grief, we cannot approach it from a “third-person perspective” and considerations deriving from such a perspective will fail to have any purchase on the experience of the sufferer (Abraham 2017, p. 16). This is because “the subjective experience in all its brutality does not allow for an objective account that can trump it in the scale of misery and pain. The first-person perspective of darkness overrides considerations that arise from a third-person perspective of light” (Abraham 2017, p. 14).

Instead, like Kilby, Abraham avers that we must recognize the limitations of theological discourse and analysis, particular when it comes to the problems of pain and suffering. What is needed, instead, is an “apophatic” approach to grief and suffering. He writes, “by apophatic I simply mean … that in this experience we are at a loss for words; we are reduced to rest and silence; we cannot say anything positive; the mystery involved ineradicable” (Abraham 2017, p. 14). Resourcing language that is traditionally used to discipline Christian speech about God, Abraham sets this method in opposition to a cataphatic approach, one which might try “to explain in positive theological terms what is happening” (Abraham 2017, p. 14).^5^ Or, to use the language of this paper, we might describe the cataphatic as an attempt to situate the mourner and their grief in relationship to God and the economy of his works. For Abraham, this desire, however motivated, must be resisted and restrained due to its inability to index the actual experience of the person-in-grief. “Any cataphatic claim has no purchase in the presence of the apophatic experience I am describing” (Abraham 2017, p. 17).^6^

For both Kilby and Abraham, grief defies theological description precisely because of the *personal* nature of grief. They both, for example, aver that grief and suffering are experienced first-personally, and theological reason requires stepping outside of the first-person to make statements from a second- or third-person vantage point. But to do so, at least with respect to grief and bereavement, is to establish a kind of distance between the subject and object of theology. So Abraham puts it, in language that echoes Kilby’s, “what happens in the deep grief of the sort I am seeking to describe is that our genuine but fragile capacities are overwhelmed; they can only deliver what emerges as a first-person perspective” (Abraham 2017, p. 16). Theological reflection upon grief would be a kind of abstraction, one that sits above and outside of the experience of grief (Kilby 2017, p. 289). But to engage in grief on these terms, as noted above, is to fail to describe and analyze grief in a manner that is commensurate with the object. Accordingly, positive descriptions of what grief are impossible, because to do so would require a kind of translation that would violate the very nature of the experience of grief. We might refer to this first tendency as the “inaccessibility of grief,” that is, the tendency to talk about grief as something that is unavailable to the “tools” theology has on offer.^7^

This acknowledged reticence to analyze grief and the phenomena associated with grief with the tools of theological reason is understandable, especially if grief’s inaccessibility proves true. Yet it is worth pausing to consider whether or not this is a given, as the authors above seem to indicate. First, it is worth noting that the primary sources of Christian theology, be they the writings of Holy Scripture or the various members of communion of the saints, do not shy away from the topics of grief, lament, and suffering. So, to take but one example, Lamentations acknowledges “For the Lord will not reject forever. Although he causes grief, he will have compassion according to the abundance of his steadfast love; for he does not willingly afflict or grieve anyone” (Lam 3:31–32). In this singular instance, we have both an acknowledgement of grief and a situating of that grief within the divine economy. Still later, the poem states, “My eyes will flow without ceasing, without respite, until the Lord from heaven looks down and sees. My eyes cause me grief at the fate of all the young women in my city” (Lam 3:49–51). Specifically, it is a grief that emerges on account of what the author is witnessing, what is happening to the people and how that affects them (Goldingay 2022, pp. 145–46). Interestingly enough, the poem puts the phenomenon of grief in a first-person perspective, written to be observed third-personally and then be appropriated first-personally (Salters 2011, pp. 11–12).^8^ This is even more interesting given the fact that it was, ostensibly, composed after the events of the exile and destruction of Jerusalem (Middlemas 2005, p. 174). Lamentations’ engagement with grief and the symptoms of grief is not unique within the biblical canon. One can look to narratives surrounding figures such as Jacob or David to see episodes of grief recorded in the third person (cf., Gen 37:34–36; 2 Sam 12:15–23; 18:33–19:4). Alternatively, one might turn to the Psalter and notice that it is filled with songs expressing grief and offering lament over the intractable problems of the world (Ps 6:6; 10:14; 31:9). And insofar as these are songs that are to be sung together,^9^ the practice of the Psalter seems to indicate, minimally, that grief can be witnessed, attested, and shared collectively (cf. Isa 22:12). In turning to the New Testament, one might note Paul’s warning against grieving the Spirit (Eph 4:30–32) or expression of “great sorrow and unceasing anguish in [his] heart” over the state of his kinsmen according to the flesh (Rom 9:2). The gospel narratives portray Christ as expressing grief over the death of his friend Lazarus (Jn 11:35–36), the status of Israel (Mt 23:37), and the prospect of his impending death (Mt 26:38). If the aforementioned writers are correct, what are we to do or make of these biblical scenes? Can any meaning be made of them that does not enact a kind of violence? It seems to me that insofar as theology, systematic or otherwise, seeks to give an account of God and his ways usward, as well as those things set in relation to God, and is principally engaged in the exposition of Holy Scripture (Webster 2012, p. 148), it is practically obligated to say something about the grief of God, however understood, and the grieving people of God.

Second, Kilby and Abraham’s emphasis on the subjective and personal nature of grief seems to elide its *social* nature. In locating grief primarily within the penumbra of the individual’s internal experience of loss, Kilby, Abraham, and Wolterstorff pass over the fact that (1) these internal experiences are only intelligible to the grieving person in light of a shared system of language and signifiers and (2) that the grieving process involves concrete actions. Regarding the former, in order to interpret and reflect upon our own experiences of grief and suffering, we must resort to our previously acquired language and concepts, both of which could be understood as a kind of sign embedded within a larger system of signs, pointing to some other experience or reality (Lacoste 2024, p. 27). But language is inherently social and necessarily shared (Spaemann 2006, p. 88), even if grief seems to push language to its breaking point. And insofar as this is the case, it seems that the experience of grief is simultaneously subjective and public (Ticciati 2022, p. 20). Furthermore, as Ferber has noted, pain, and grief as a species of that larger genus, not merely isolates us from others and turns us in on ourselves but also deepens our bonds with others (Ferber 2019, p. 146).

Moreover, it is perhaps not helpful to conceive of grief as a mere subjective set of experiences. For those experiences themselves are bound up with the process of grieving. Here, the work of clinical psychologists Margaret Stroebe and Henk Schut explain how bereavement entails two components. Their dual process model of grief proposes that there are “two categories of stressors associated with bereavement, namely, those that are loss- versus restoration-oriented” (Stroebe and Schut 2010, p. 277). Grief is not merely “loss-oriented,” but also entails an orientation toward a future recovery. For example, while Adeodatus mourns the death of his grandmother, he must also begin to engage in specific tasks that contribute to imagining a new life, post-loss. He must figure out how all the formerly shared tasks will now be distributed: who will file taxes, who will shop for groceries and prepare meals, etc. Stroebe and Schut refer to these as “restoration-oriented stressors” (Stroebe et al. 2005, p. 50). In grieving the loss of a loved one, people frequently oscillate between periods of focusing on the loss and focusing on restoration and recovery (Stroebe and Schut 2010, p. 276) (For an overview, see Stroebe and Schut 1999, pp. 197–224). For Stroebe and Schut, grief involves “a dynamic coping process, namely, a regulatory process labeled *oscillation* … the principle underlying oscillation is that at times the bereaved will confront aspects of loss, at other times avoid them, and the same applies to the tasks of restoration” (Stroebe and Schut 2010, p. 278). And these shifts are not happenstance but are often the result of intentional thoughts and choices (Stroebe and Schut 2010, p. 277). Insofar as this is the case, grief is not reducible to the “feeling” of loss or the disorientation that accompanies loss, however eliding of explication this feeling can often seem to be. Moreover, the expressions of grief and actions that are bound up with the grieving process are invariably social in nature and communicative of particular desires, needs, priorities, intentions, and concerns. And if, as Oliver O’Donovan explains, communication involves the inhabitation of “a shared structure of meanings,” which suggests that observing the communicative actions of the grieving process may provide us with grounds for theologically describing the dialectic of grief, moving back and forth from focusing on the loss and the pain associated with that loss to focusing on life as it is now encountered (O’Donovan 2017, p. 48).

## 3. The Study and Science of Grief

### 3.1. Observing Grief: The Psychosomatic Expressions of Grief and Bereavement

Although the discipline has developed and changed over time, psychological scientists, since the turn of the 20th century, have turned their attention to the nature of grief, making it the objective of empirical investigation (Granek 2010, pp. 46–73). Grief, as a psychological concept, refers to “the emotional reaction to the loss of a loved one that can include sadness, longing, sorrow, despair, and anguish” (Granek 2010, p. 47). Here grief, as the emotional reaction to or distress one feels as a result of losing a loved one, is differentiated from grieving, the process by which an individual or a group of individuals copes with and adapts to the loss of a loved one, over time, and bereavement, the period of time after someone dies that someone is grieving. Insofar as psychological scientists view grief, grieving, and bereavement as concepts accessible to empirical investigation, they view such concepts as elements of human life that can be observed in human behavior, analyzed, engaged, predicted, and understood (Danziger 2003, pp. 20–21).

First, and importantly, insofar as psychological science subjects grief to empirical research, psychological scientists have found that the experience of grief is observable in human behavior, physiology, and neurobiology (Holinger 2020, pp. 39–43). Grief is observable in symptoms such as longing for the deceased, persistent emotional pain, difficulty accepting the loss, or avoiding reminders of the deceased. These symptoms can be expressed in behaviors such as seeking out the deceased (longing for the deceased), crying (emotional pain), or refusing to enter the study or office of a loved one who has died (avoiding reminders). We can, then, identify people who are grieving and responding to loss based on their behavior.

Researchers have also found that grief is observable in human physiology and biomarkers. So, for example, Christopher Fagundes et al. conducted a study to better understand the effects of bereavement on a population of widows and widowers. They found that the heart rate variability of the bereaved was lower than that of their age-matched comparisons, indicating that they were at higher risk for cardiovascular disease. Moreover, Fagundes et al. measured the level of cytokiness, a category of small protein produced by a broad range of immune cells, within the bereaved with respect to a non-bereaved control group in order to assess the health and activity of their immune system. Higher levels of cytokines are associated with heightened levels of inflammation. The study found that “those who were bereaved had significantly higher levels of the composite stimulated cytokine profile compared with those who were not bereaved” (Fagundes et al. 2018, pp. 68–69). In other words, for those experiencing bereavement, neither the cardiovascular system nor the immune system is functioning as optimally as the cardiovascular and immune systems of the non-bereaved.

On these grounds, researchers are also able to distinguish between people’s variable responses to loss, that is, between so-called “normal grief” from “complications to the grieving process.” Researchers will use measures such as the Texas Revised Inventory of Grief-Present Scale and the Complicated Grief Inventory or the Prolonged Grief Disorder—13 scale in order to differentiate the two psychological constructs. “Normal grief” is characterized by occasionally missing the deceased, feeling the need to cry at times, and being reminded of the deceased. “Complicated grief,” by contrast, is characterized by symptoms such as an intense longing for the deceased, numbness and detachment, feelings of emptiness, and chronic and disruptive feelings, among other things, that are present within the bereaved for longer than two months (Boelen and van den Bout 2008, p. 313; Dillen et al. 2008, p. 392). Observing the duration, intensity, and recurrence of grief related symptoms, then, provides some insight into the nature of an individual’s bereavement. And insofar as grief is observable in behavior, physiology, and biomarkers, and insofar as grief is classifiable into particular forms or kinds, it seems that grief is not so internal as to be rendered inaccessible to rational analysis from a third-person perspective even if such analysis cannot hope to be comprehensive or totalizing.

### 3.2. Grief, Attachment Theory, and the Experience of Bereavement

Psychological engagement with the phenomenon of grief has not merely observed and disaggregated but also sought to understand the nature of this grief with respect to human relationality, for again, the actions and processes associated with grief are invariably social in nature. Recent work in the psychology of grief has drawn on the attachment theory to explain the dysregulating nature of bereavement. Attachment theory focuses on the manner in which human beings are born with an innate system that encourages them to seek relationships with others that will provide support, protection, and consolation in times of crisis (Mikulincer and Shaver 2022, p. 1). While initially developed as a method for understanding infant-caregiver relationships and bonds (Bowlby 1969; Ainsworth et al. 1978). More recently, see (Hofer 1996, pp. 570–81), the theory has been expanded to include the various relationships that adolescents and adults form as they mature and age (see, for example, Hazan and Shaver 1987, pp. 511–24). According to Feeney, a particular figure in, say, Adeodatus’ life can be characterized as an “attachment figure” insofar as Adeodatus seeks proximity to them, resists separation from them, turns to them in stress, and resources them as a secure base for interacting with the world (Feeney 2004, pp. 631–32). These attachment figures are vital for successfully navigating the various stressors of life, assist in the regulation of biological systems, and confer positive health benefits such as the reduction in cardiovascular arousal and enhanced longevity.^10^ Additionally, attachment relationships are important for assisting in the regulation of each member’s emotional system (Coan et al. 2006, pp. 1032–39). As Sbarra and Hazan put it succinctly, “Humans need social ties to function best” (Sbarra and Hazan 2008, p. 142).

The concept of “attachment hierarchies” helps explain the nature and intensity of an individual’s different attachment relationships (Bretherton 1985, p. 33). The locution “attachment hierarchy” refers to “one’s collection of others arranged according to whom the individual prefers to orient toward for various components of attachment” (Trinke and Bartholomew 1997, p. 604). Most people have multiple attachment figures throughout the course of their life and “selectively orient attachment-related functions toward one primary attachment figure, as well as a number of secondary attachment figures” (LeRoy et al. 2019, p. 392). So, Adeodatus may be attached to both his grandmother, Monica, and his neighbor, Alypius, but the former may play a more significant role in comforting or encouraging him, especially in times of significant stress. As Rosenthal and Kobak note, “a primary attachment figure can be identified from a child’s preferences for that person in situations that test the presence of an attachment bond” (Rosenthal and Kobak 2010, pp. 681–82). Insofar as this is the case, Monica would occupy a “higher” place in Adeodatus’ attachment hierarchy. Importantly, these attachment hierarchies are not static, but dynamic and must be renegotiated and reorganized as an individual ages and develops (Fraley and Davis 1997, pp. 131–44). So, if Adeodatus were to find a romantic partner, this may necessitate the reorganization of his attachment hierarchy so that the partner now serves as his primary attachment figure.

The notion of attachment hierarchies provides a helpful interpretive key for understanding the nature of bereavement. Field et al. found that continued attachment to a deceased partner five years after their death was related to the perdurance of feelings of intense distress (Field et al. 2003, pp. 110–17). In the event of the death of a loved one near the top of that attachment hierarchy, the bereaved experience a kind of dysregulation of their biological systems. As LeRoy et al. (LeRoy et al. 2019, p. 392), put it, when someone is bereaved of a primary attachment figure “an ‘empty’ space is created at the top of the surviving partner’s attachment hierarchy. Accordingly, there is no one available to whom the surviving partner can effectively direct his or her attachment-related needs”. This loss results in the experience of the disruption and dysregulation of their homeostasis, evidenced in a dysregulated immune system, a higher risk of depression, and elevated cardiovascular risk (Fagundes et al. 2018, pp. 65–71). Additionally, while not in the context of bereavement, Fagundes found that individuals who viewed their former partner as their principal attachment figure reported more intense feelings of distress than those who no longer felt the need to seek out a lost partner for various forms of support (Fagundes et al. 2018, pp. 37–50).

The experience of bereavement of those to whom we are sufficiently attached can be particularly jarring as these attachment relationships help construct and stabilize our sense of self. An individual’s attachment relationship or relational role as, say, father, mother, sister, etc., is not merely a role, they are relationships and our sense of self exists only within these kinds of relationships. The death of a loved one can result in a change in identity and roles for the bereaved (Stroebe et al. 2005, p. 51). Accordingly, in the event of the death of someone to whom an individual is closely attached, then, they not only need to engage in the process of reorganizing their attachment hierarchy but also need to rebuild their sense of self. So, it is perhaps unsurprising that researchers have found an association between interpersonal closeness and the intensity and duration of the bereaved partner’s grief. For example, after controlling for the severity of depressive symptoms and other loss-related variables, Sekowski and Prigerson sought to better understand the relationship between emotional dependency and prolonged grief disorder. In their study, they found “a significant positive association between emotional dependency and [Prolonged Grief Disorder],” in the bereaved after the death of a spouse or loved one (Sekowski and Prigerson 2021, p. 4). Indeed, several studies have found that those suffering from prolonged grief disorder viewed their identities as closely connected with the deceased (See Bellet et al. 2020, pp. 543–43; Boelen 2012, pp. 117–24; Maccallum and Bryant 2008, pp. 1311–15; Robinaugh and McNally 2013, pp. 290–300). A study conducted by Harrison et al. found that “the perceived interpersonal closeness of the bereaved with the deceased after death was a statistically significant predictor of [Prolonged Grief Disorder]” (Harrison et al. 2022, p. 1107). That is to say, the more closely Monica and Adeodatus are to one another and the more they depend upon one another, the greater their risk of experiencing a severe yearning and longing for one another in the event of bereavement as well as a sense of emptiness in life apart from them.

Two insights are worth noting from this attenuated overview of the psychological literature. First, it seems that insofar as grief is observable in human behavior, physiology, and biology and apparent to the degree that it can be classified and analyzed, then it is not totally inaccessible to rational analysis. It seems to me that Kilby, Abraham, and Wolterstorff may overly internalize the experience of grief. We grieve in, with, and through our bodies, biologically and behaviorally (Bavinck 2018, p. 101). And insofar as we are situated in relationship with and to other bodies, and the body is expressive of the person, we grieve with and toward other persons. Second, and importantly, it seems to me that the language of attachment and bonds, in conjunction with the role these attachments play in shaping our sense of self, provides a framework for understanding what is being grieved and why the pain of loss is felt so acutely. We grieve because we are attached, and we are attached because we love. And insofar as this is the case, it seems to me that there are grounds for thinking theologically about the phenomenon of human grief and attachment, even if such reflection must be incomprehensive and paradoxical in nature.

## 4. Theological Fragments of Grief

So, it seems to be the case that we can see and observe grief and that the process of grieving is invariably social in nature, bound up with language, both verbal and non-, and social practices that express the “heart” of the person. But this does not require, as Kilby and Abraham both have warned, an assertion of the goodness of grief and suffering, let alone an attempt to account for its normative significance in the life of a particular person. Rather, what it affords us is the opportunity to describe grief theologically and to situate it as a unique feature of the saeculum. Insofar as the foregoing is correct, we might offer the following working theological description of grief: *grief is the experience and process of being confronted with and recovering from the various forms of the prolepses of death within the postlapsum world, especially pertaining to the disintegration and reintegration of the “generational self” as it witnesses and heals from the wounds left by the loss(es) of those to whom we are deeply attached*. In light of that description, we can unpack two “fragments” of grief: grief as the disintegration and reintegration of the pilgriming, postlapsum self.

### 4.1. Grief and the Disintegrating Self

Human life unfolds in generations and relationships across time (Radner 2016, p. 116). Existing in relations of unconditional dependence and contingency upon their Creator (Isa 40:12), while also subsisting in relationships with other creatures, human creatures exist as embodied entities, extended here or there, now or then. And insofar as this is the case, these bodies are set off from, next to, and in relationship with other bodies (Sonderegger 2016, pp. 391–92). More might be said here. Human creatures not only exist in relationships with other creatures in virtue of their proximity to or causal dependency upon them, but also are situated in relationships of family, friendship, peoplehood, etc. These latter relationships are extended across time in the form of generations. “O God, our ancestors have told us,” the Psalmist avers, situating themselves and subsequent singers of the Psalm with respect to generations gone past (Ps 44:1).^11^ Human life unfolds “*from* generation to generation” (Lk 1:50), but also *in* generations (cf. Gen 6:9) and *toward* future generations (cf. Gen 9:12; Ex 34:7). Insofar as human life is situated within relationships and from, in, and toward generations, so too is the identity and self-understanding of those human beings. That is to say, our identities do not exist or unfold in abstraction from the concrete persons from whom we receive our life and the other persons we connect with, deeply attach ourselves to, and love.

In many ways, we grieve “because of our ties to other people as well as our ties to our own past and future selves” (Cholbi 2022, p. 11). To the extent that human life not only unfolds in generations and relationships over the course of a “span” or lifetime (Marías 1971, p. 77; cf. Ps 90:10), when a loved one dies, we not only experience their absence but also the loss of the future life we imagined and expected to experience with them. Insofar as the futures we pursue order and explain the motivations for our actions, these imagined futures also play a significant role in expositing our sense of self. I am, in many ways, the creature who lives and acts now, but my actions are founded on intentions, a horizon I act toward and hope to realize (Spaemann 2006, pp. 54–55). But these future events and the goods pertaining thereto invariably involve other people: our loved ones, our family members, our friends, etc. We, in other words, anticipate particular future events, and these future events invariably include the participation of particular individuals. Anticipation, as Oliver O’Donovan points out, is a mode of relating to the future and the expectation of the realization of potential futures. He writes, “anticipation teases out a future that lurks within the present as a possibility” (O’Donovan 2013, p. 121). For O’Donovan, anticipation is based on present, probability and patterns of perception (O’Donovan 2013, p. 121). So, to adapt an example from O’Donovan, Adeodatus looks into a handful of pear seeds and envisions the future plants that will sprout from them. He and his grandmother, Monica, plant the seeds in the ground, having fertilized the soil and provided sufficient water, expecting the seeds to germinate, grow into trees, and then produce buds that will eventually flower. This “potential” future lurks inside the nature of the pear seed. Adeodatus and Monic would not, however, anticipate that these pear saplings will sprout legs or develop gills in time, because, ostensibly, such futures are not inherent or intrinsic to the nature of pear seeds as such. Anticipation is a disposition toward the future that is rooted in the possibilities of the present, a fact O’Donovan uses to distinguish anticipation and hope.

This provides us with grounds for returning to address Strawson’s claim about grief and death, which he ties explicitly to our relationship to future possibilities (Strawson 2018, pp. 75–77). Yet while on Strawson’s understanding such a future world does not objectively belong to us, thereby enabling us to identity the dissolution of a possible future as a genuine loss, O’Donovan’s reflections on anticipation provide a helpful counterpoint point, and perhaps can be augmented to make a stronger claim with help from Julián Marías. According to Marías, human beings inhabit two worlds simultaneously: the present world and the world of their imagined future. He writes, “we live primarily in the future. I am not future … but perfectly real and present … I am [also] *futurizo*—present, but oriented toward the future, turned toward it, projected toward it. I am in *this* world and in the *other*: the world I anticipate, project, imagine, the world that is not there, the world of tomorrow. And this world, the world of my plans … is the world that confers its worldhood—its character as a world—on this material and present world” (Marías 1971, pp. 14–15). For Marías, the person is always looking toward the future and without this future, our lives become unintelligible. Monica wakes up in the morning, in view of what she will do that afternoon and the next day, whether it is harvesting pears or conversing with bishop Ambrose. “Human life,” on Marías’s account, “operates essentially in anticipation of the future (Marías 1971, p. 39). If Marías is correct, insofar as anticipation views a future possibility as intrinsic to the object (e.g., the realization of pear tree from a pear seed), when an “other” is incorporated into our self and we anticipate futures with them (e.g., attending graduation; participating in a martial ceremony), we live in and toward that world. Importantly, these futures are bound up with these “incorporated others” and thereby become intrinsic to our sense of self. So, Adeodatus anticipates watching the ships come into the dock with Monica and pruning the trees in their pear orchard together.

This vision of future events exists as a “germ,” to borrow language from Friederich Schleiermacher’s Christology. When that “other” dies and we experience bereavement, we mourn not only the loss of the other and experience a kind of damage to the self, but we also mourn the death of that “germ,” those anticipated futures that necessarily involved the departed other (Nussbaum 2001, p. 83). So, for example, when Monica imagines her future retirement reception, Adeodatus plays a necessary role in that portrayal, perhaps in giving a speech or offering a tribute. If he were to die prematurely and tragically, this future vision dies with him. This coheres well with recent research into the phenomenon of grief and bereavement, especially as it relates to the loss parents experience with respect to the untimely death of their children. As Michael McNeil et al., note, “Parents grieve not only the death of their child but also the loss of their future hopes and plans for the child and family” (McNeil et al. 2021, p. 2). Accordingly, Monica may be able to look forward to her retirement party again, one day, but this envisioned future would be, properly speaking, a *novum* and insofar as she does this, she is engaging in the process of reconstructing and reintegrating her “selfhood.”

### 4.2. Grief and the Reintegrating Self

Yet, as noted above, we must not reduce grief and the grieving process to solely the disintegrated self and the death of future possibilities. Human beings are relational creatures with fundamental needs (McKirland 2022, pp. 23–26). If we are to carry on and continue living in the face of bereavement, the needs and roles fulfilled by those who have died must be attended to. Some philosophers have objected to this resilient commitment to continuing on in the face of loss and bereavement, worrying that it is unethical and renders us unable to attend to the nature of our condition (Moller 2007, pp. 310–11). As Marušić points out in his reflections on grief and anger, the very reasons that often enable us to move forward in processing these emotions would be morally repugnant to us in the early stages of grief (Marušić 2018, p. 3). Yet as time elapses, we become, in some ways, detached from the objects of grief (Marušić 2022, p. 95). So it seems to us that even while grief leaves a kind of damage or death to the self and “my world,” it also leads to the transformation of the self (Markovic 2024, p. 250). This too is a part of the experience of grief. In other words, grief also indexes the process of rebuilding, both of our lives and our senses of self, in response to the death of a loved one to whom we are deeply attached (Stroebe and Schut 2010, p. 278). While “weeping may linger for the night … joy comes in the morning” (Ps 30:5). Yet importantly, on Augustine’s reading, the joy in question does not consist in the abnegation of loss or the removal of the memory of the one who has died and morning does not refer to the subsequent day. Rather, humanity exists “in the evening … [in] a prolonged period of weeping” (Augustine 2000, p. 298). For Augustine, the “joy” of the coming morning is then reserved for the absolute future that God has promised to bring about, a joy that is experienced in the resurrection of the body and the life everlasting. Nevertheless, while the fullness of joy in a theological sense lies off in the eschatological future with the hope of resurrection, a point Augustine is at pains to acknowledge, our mortal lives unfold in the present epoch of “the evening,” a time of grief. He writes, “Lament for the things of the present, sing of what is to come in the future … you must look forward in hope to the morning and be joyful in it; but for the present you have to grit your teeth and lament” (Augustine 2000, pp. 311–12). Augustine provides his own version of Stroebe and Strut’s oscillation, albeit one that is more focused on ecclesial practices such as prayer, praise, and lament. For Augustine, we oscillate between singing in expectation of the coming joy while we lament in present for our current experience of grief. We can look forward in anticipation, while enduring and expressing the pains of the present. And insofar as this is the case, this oscillation involves the process of rebuilding and reintegrating the wounded self in the wake of loss so that it might endure the world.

In grief, we make intentional choices: choices to pay bills, pick the kids up from school and take them to choir rehearsal, and cancel credit cards. These actions involve the care for the various goods pertaining to mortal lives (Radner 2024, p. xxiii). Indeed, as noted above, those grieving the loss of a loved one do not typically “wait” until they are no longer experiencing bereavement to make these choices, but oscillate between focusing on the present loss and focusing on the tasks of restoration and recovery (Stroebe et al. 2005, p. 52). These recovery-oriented choices and tasks are actions still undergone as a result of and in response to the loss of a loved one, but they are also actions which begin to formulate and construct a particular future that we are beginning to imagine, a future that will render these actions coherent (Marías 1971, p. 80). That is, while Adeodatus may feel that he is not able to imagine life without Monica, in choosing to engage in “recovery oriented activities” he is beginning to do just that. And insofar as this is the case, in the choices Adeodatus executes in orientation to the recovery of grief he begins to reformulate his identity, he begins to form an answer to the question: who am I without this person?

In bereavement, the human subject works to formulate an answer to this question, to adjust to a radically reconfigured understanding of the world. The behaviors undergone here are not inherently irrational or illogical. They are actions that must be understood with respect to the specific wounds the self has received, the state of “being-wounded” in which it exists, the world the self-inhabits post-loss, and the steps required to reintegrate and recover from those wounds. Here it is helpful to think of bereavement as a kind of catastrophe that has overturned the world of the bereaved.^12^ With the locution “the world,” I refer to what each of us makes of the fluid and changing circumstances surrounding our lives.^13^ In the death of a loved one, especially one to whom we are sufficiently attached, this world is radically altered and the disruption of our attachment relationships is catastrophic. Nevertheless, the intentional choices made in the wake of bereavement (e.g., paying bills in the case of Adeodatus; prayer and lament in the case of Augustine) are actions that make perfect sense from within the context of a new, overturned world, from within the context of a world that needs to be remade. That is, these actions can be understood as a kind of renegotiation of or accommodation to a new reality, even as we strive to envision ourselves anew in a post-loss world. And it is this selfsame “overturned world” that must be endured.^14^ Grieving, then, can be understood as the process of enduring the wounds the self has received in loss even as we attempt to rebuild and reconstruct a life in the wake of bereavement. And insofar as this is the case, and our understandings of “the world” lie intrinsic to our senses of self and our hoped-for futures, then in the wake of loss we are trying to reconstruct and reintegrate the wounded self. In the context of a devastated world, we are consistently trying to negotiate the wounds of bereavement, care for its attendant damage, and reimagine a life on the other side of catastrophe.

## 5. Conclusions

Theological engagement with grief is a theology in and of the saeculum, the time between Christ’s first and second advents, surrounding the negotiation of the penultimate and mortal goods we pursue, attach ourselves to, lose, and lament over within that context. Accordingly, there must be significant but not absolute restraint when it comes to the issue of positing the value of grief, mourning, and suffering in the world, as point Kilby and Abraham rightly note. Yet, as we have argued above, this restraint does not equate to a kind of apophatic silence on the nature of grief, the reasons we grieve, and where grief and the griever are situated within the economy of God’s works. Insofar as grief is an observable and shared phenomenon, it exists as the object of both psychological *and* theological consideration. That is to say, people grieve because of their situatedness within relationships in the present age of suffering and this grief is expressed in their behavior and in their physiology. Of course, the story of Holy Scripture concludes with the powerful image of the Lord God promising to bring about an end to grief and the process of grieving alike. The Lord promises that “he will wipe every tear from their eyes. Death will be no more; mourning crying and pain will be no more, for the first things have passed away” (Rev 21:4). But human lives unfold within the interim, “in the time of weeping,” as Augustine puts it, and these lives are shaped, warped, and formed by grief (Augustine 2000, p. 288). And if this is the case, it seems to me that theological resources must be brought to bear on describing the phenomenon, but in a way that resists the temptation to ascribe or identify the goods resultant from grief in and for the present. And so we might speak of grief “in fragments,” and perhaps only so. If this is the case, it seems to me that theological resources must be brought to bear on describing the phenomenon, but in a way that resists the triumphalist temptation to ascribe or identify the goods resultant from grief.

## Data Availability

No new data were created or analyzed in this study. Data sharing is not applicable to this article.

## Footnotes

1 This is, of course, a broad generalization and attempts at theodicy continue to proliferate (see, for example, Motto 2024, p. 1149; Peckham 2018; Vitale 2023; Sollereder 2020; Pak 2016).

2 This may be why some theologians or theologically inclined philosophers have shifted from offering a theodicy per se, to offering defenses of God’s goodness in the light of suffering and evil. Stump writes, “What distinguishes a defense from a theodicy is that a defense does not claim that the possible world it describes is the actual world … makes no claims about the way the actual world is or about any actual intentions and reasons for allowing evil on the part of God” (Stump 2012, p. 19). Stump’s distinction between a theodicy and a defense of God, builds off a distinction that is helpfully clarified in the work of figures such as Peter van Inwagen. The difference between a theodicy and a defense, on van Inwagen’s reading, “lies not in their content but in their purposes. A theodicy is a story that is told as the real truth of the matter; a defense is a story that, according to the teller, may or may not be true” (van Inwagen 2006, p. 7). Still, for both van Inwagen and Stump, it is at least conceptually possible that the story told in a defense of God’s goodness in light of present suffering turns out to be true (van Inwagen 1991, p. 141). Accordingly, a defense aims at plausibility; not actuality or certainty. For a similar refusal to submit the problems of evil and suffering to rational analysis, theological or otherwise, see Burrell 2008).

3 Here, we are appropriating the distinction between the descriptive and the normative task of Christian theology as articulated in Fellipe Do Vale’s *Gender as Love*. Do Vale argues that frequently, accounts of gender fail to have any purchase on life as we live it and the social goods we must navigate therein. He proposes that we should instead think of the theological task, as it relates to theologies of gender, as two-fold: “first, they must be *descriptively* accountable, making sense of the gendered lives we already live. What are the gendered goods encountered by the individuals in these contexts?…Second, they must be *normatively* accountable, telling us not just what gender *is* but what it *ought* to be, or how to engage these goods *rightly*, as we encounter them simply by living our lives…The normative task is not completed by providing definitions of gender that are not descriptive. Rather, the normative task must take into account the descriptions already provided and work *within those* to love rightly” (Do Vale 2023, pp. 165–66). In what follows, as we will see below, we will attempt to resource psychological science to provide a salient theological *description* of grief.

4 In a footnote Kilby adds an important clarification: “I am not advocating the assertion of *logically* incompatible propositions, but rather the holding of a set of beliefs which, somewhat more broadly, *we cannot make sense of*. There may be some other perspective in which they all make sense together, but if so this is something of which we cannot even begin to conceive” (Kilby 2020a, p. 82).

5 Kilby too resources this distinction. She writes, “some echo, some analogy to the cataphatic/apophatic dialectic is, we think, in fact present in our relationship to suffering” (Kilby 2020b, p. 100).

6 A similar description of the effect of grief is offered by Nicholas Wolterstorff as he reflects upon the death of his Son, as originally recorded in his book *Lament for a Son*. He too describes grief as disruptive his cognitive and analytic powers. Wolterstorff, like Abraham, found little solace in the answers traditional theodicies provide, especially as it pertains to the problem of evil and suffering in the world. Instead, he finds that recourse to the mysterious nature of God and his ways usward is the only option left available to a kind of apophatic silence, although he does not use this language explicitly. He writes, “Rather than Eric’s death evoking in me an interest in theodicy, it had the effect of making God more mysterious. I live with the mystery” (Wolterstorff 2019, p. 29).

7 Here we might see a parallel to how 20th century philosophy of language tended to speak of pain as unraveling discursive processes, especially insofar as they are dependent upon linguistics. Ferber’s analysis on this point is especially apt, writing, “The paradigms that construe pain’s encounter with language as being overwhelmingly destructive and isolating are both grounded, strongly but also blindly, on criteria of success or failure. From such a perspective, pain cannot be described, referred to, or fully communicated; language, whether propositional or performative, does not furnish a way out of pain; it cannot relieve or alter it. In this sense language cannot perform in the face of intense pain, presenting us with a linguistic abyss” (Ferber 2019, p. 147).

8 Koenen notes that it seems as if the poems of Lamentations were written to be read for people to listen to and to indirectly facilitation their expression of grief to Israel’s God (Koenen 2015, pp. 17, 92). Insofar as this is the case, the poems are written to invite a communal expression of grief and thereby indicate a kind of communicability with respect to the poem’s author, the reader, and the members of the liturgical community.

9 Tremper Longman III writes, “The primary use of the book of Psalms, the literary sanctuary, during the Old Testament period was in the public corporate worship of Israel. Psalms has rightly been called the ‘Hymnbook of the Old Testament’” (Longman 2014, p. 36).

10 On the relationship of attachment to health outcomes, in general, see, (Robles and Kiecolt-Glaser 2003, pp. 409–16). On the relationship of attachment to cardiovascular arousal see, (Grewen et al. 2003, pp. 123–30). On the relationship of attachment to enhanced longevity see (Rohrbaugh et al. 2008, pp. 781–89).

11 Ross notes that the language here is intended to link the audience and Psalmist to “a long line of traditon going back” to previous events (Ross 2011, p. 45).

12 We are borrowing the language of catastrophe from Ephraim Radner. Radner writes, “catastrophe, in a Christian sense, is not about endings but about the character of the ‘middle,’ which lies between unsought beginnings and inevitable endings. That middle is, of course, characterized by a host of smaller, if often wrenching, endings” (Radner 2024, p. 129).

13 Marías describes “our world” as the “circumstances surrounding our lives,” one that “is composed of changing situations whose ingredients enter and exit” (Marías 1971, pp. 97, 102).

14 Here, we are using language from Charles Mathewes *A Theology of Public Life*. He writes, “To endure our life … is to be attentive and wakeful, patient and long-suffering, to refuse to let the world have the last word on what it means, and yet to refuse to presume to know what that last word will be. It is to live in the world, without accepting its immanent self-presentation” (Mathewes 2007, p. 15). While Matthewes is speaking of Christian political participation in “public life” and uses it with less phenomenological implications, we believe that some translation is possible from “the world” to “my world,” especially in the context of grief.

## References

[R1] Abraham William J. 2017. Among the Ashes: On Death, Grief, and Hope. Grand Rapids: Eerdmans.

[R2] Ainsworth Mary D. Salter, Blehar Mary C., Waters Everett, and Wall Sally. 1978. Patterns of Attachment: A Psychological Study of the Strange Situation. Mahwah: Lawrence Erlbaum.

[R3] Augustine of Hippo. 2000. Expositions of the Psalms: Volume 1. Translated by Boulding Maria. The Works of Saint Augustine: A New Translation for the 21st Century II/15. Hyde Park: New City Press.

[R4] Bavinck Herman. 2018. “Foundations of Psychology.” Translated by Born Jack Vanden, Kloosterman Nelson D., and Bolt John. Bavinck Review 9: 1–252.

[R5] Bellet Benjamin W., LeBlanc Nicole J., Nizzi Marie-Christine, Carter Mikaela L., van der Does Florentine H. S., Peters Jacqueline, Robinaugh Donald J., and McNally Richard J.. 2020. ‘Identity Confusion in Complicated Grief: A Closer Look’: Correction. Journal of Abnormal Psychology 129: 543–43. 10.1037/abn0000631 32551740

[R6] Boelen Paul A. 2012. A Prospective Examination of the Association between the Centrality of a Loss and Post-Loss Psychopathology. Journal of Affective Disorders 137: 117–24. 10.1016/j.jad.2011.12.004 22240087

[R7] Boelen Paul A., and van den Bout Jan. 2008. Complicated Grief and Uncomplicated Grief Are Distinguishable Constructs. Psychiatry Research 157: 311–14. 10.1016/j.psychres.2007.05.013 17916387

[R8] Bowlby John. 1969. Attachment and Loss. 3 vols. New York: Basic Books.

[R9] Bretherton Inge. 1985. Attachment Theory: Retrospect and Prospect. Monographs of the Society for Research in Child Development 50: 3–35. 4069128

[R10] Burrell David B. 2008. Deconstructing Theodicy: Why Job Has Nothing to Say to the Puzzle of Suffering. Grand Rapids: Brazos Press.

[R11] Cholbi Michael. 2022. Grief: A Philosophical Guide. Princeton: Princeton University Press.

[R12] Coan James A., Schaefer Hillary S., and Davidson Richard J.. 2006. Lending a Hand: Social Regulation of the Neural Response to Threat. Psychological Science 17: 1032–39. 10.1111/j.1467-9280.2006.01832.x 17201784

[R13] Danziger Kurt. 2003. Where History, Theory, and Philosophy Meet: The Biography of Psychological Objects. In About Psychology: Essays at the Crossroads of History, Theory, and Philosophy. Edited by Hill Darryl B. and Kral Michael J.. Alternatives in Psychology. Albany: State University of New York Press.

[R14] Dillen Let, Fontaine Johnny R. J., and Verhofstadt-Denève Leni. 2008. Are Normal and Complicated Grief Different Constructs? A Confirmatory Factor Analytic Test. Clinical Psychology & Psychotherapy 15: 386–95. 10.1002/cpp.590 19115457

[R15] Do Vale Fellipe. 2023. Gender as Love: A Theological Account of Human Identity, Embodied Desire, and Our Social Worlds. Grand Rapids: Baker Academic.

[R16] Fagundes Christopher P., Murdock Kyle W., Angie LeRoy Faiza Baameur, Thayer Julian F., and Heijnen Cobi. 2018. Spousal Bereavement Is Associated with More Pronounced Ex Vivo Cytokine Production and Lower Heart Rate Variability: Mechanisms Underlying Cardiovascular Risk? Psychoneuroendocrinology 93: 65–71. 10.1016/j.psyneuen.2018.04.010 29702444

[R17] Feeney Brooke C. 2004. A Secure Base: Responsive Support of Goal Strivings and Exploration in Adult Intimate Relationships. Journal of Personality and Social Psychology 87: 631–48. 10.1037/0022-3514.87.5.631 15535776

[R18] Ferber Ilit. 2019. Language Pangs: On Pain and the Origin of Language. Oxford: Oxford University Press.

[R19] Field Nigel P., Gal-Oz Eval, and Bonanno George A.. 2003. Continuing Bonds and Adjustment at 5 Years after the Death of a Spouse. Journal of Consulting and Clinical Psychology 71: 110–17, Scopus. 10.1037//0022-006x.71.1.110 12602431

[R20] Fraley R. Chris, and Davis Keith E.. 1997. Attachment Formation and Transfer in Young Adults’ Close Friendships and Romantic Relationships. Personal Relationships 4: 131–44.

[R21] Goldingay John. 2022. The Book of Lamentations. New International Commentary on the Old Testament. Grand Rapids: Eerdmans.

[R22] Granek Leeat. 2010. Grief as Pathology: The Evolution of Grief Theory in Psychology from Freud to the Present. History of Psychology 13: 46–73. 10.1037/a0016991 20499613

[R23] Gregory of Nyssa. 2005. The Life of Saint Macrina. Translated by Corrigan Kevin. Eugene: Wipf and Stock.

[R24] Grewen Karen M., Anderson Bobbi J., Girdler Susan S., and Light Kathleen C.. 2003. Warm Partner Contact Is Related to Lower Cardiovascular Reactivity. Behavioral Medicine 29: 123–30. 10.1080/08964280309596065 15206831

[R25] Harrison Octavia, Windmann Sabine, Rosner Rita, and Steil Regina. 2022. Inclusion of the Other in the Self as a Potential Risk Factor for Prolonged Grief Disorder: A Comparison of Patients with Matched Bereaved Healthy Controls. Clinical Psychology & Psychotherapy 29: 1101–12. 10.1002/cpp.2697 34822735

[R26] Hazan Cindy, and Shaver Phillip. 1987. Romantic Love Conceptualized as an Attachment Process. Journal of Personality and Social Psychology 52: 511–24. 10.1037//0022-3514.52.3.511 3572722

[R27] Hofer Myron A. 1996. On the Nature and Consequences of Early Loss. Psychosomatic Medicine 58: 570–81. 10.1097/00006842-199611000-00005 8948005

[R28] Holinger Dorothy P. 2020. The Anatomy of Grief. New Haven: Yale University Press.

[R29] Julian of Norwich. 2015. Revelations of Divine Love. Translated by Windeatt Barry. Oxford World’s Classics. Oxford: Oxford University Press.

[R30] Kilby Karen. 2017. Eschatology, Suffering and the Limits of Theology. In Game Over?: Reconsidering Eschatology. Edited by Chalamet Christophe, Dettwiler Andreas, Mazzocco Mariel and Waterlot Ghislain. Theologische Bibliothek Töpelmann 180. Berlin: De Gruyter, pp. 279–91.

[R31] Kilby Karen. 2020a. God, Evil and the Limits of Theology. London: T&T Clark.

[R32] Kilby Karen. 2020b. Negative Theology and Meaningless Suffering. Modern Theology 36: 92–104.

[R33] Kilby Karen. 2020c. The Seductions of Kenosis. In Suffering and the Christian Life. Edited by Kilby Karen and Davies Rachel. London: T&T Clark, pp. 163–74.

[R34] Koenen Klaus. 2015. Klagelieder (Threni). Biblischer Kommentar Altest Testament—Neubearbeitungen, 20. Göttingen: Vandenhoeck & Ruprecht Gmbh & Co.

[R35] Lacoste Jean Yves. 2024. In Search of Speech: Talking, Reading and Praying in an Age of Words. Translated by O’Donovan Oliver. London: Bloomsbury.

[R36] LeRoy Angie S., Knee C. Raymond, Derrick Jaye L., and Fagundes Christopher P.. 2019. Implications for Reward Processing in Differential Responses to Loss: Impacts on Attachment Hierarchy Reorganization. Personality and Social Psychology Review 23: 391–405. 10.1177/1088868319853895 PMC6896215 31200625

[R37] Longman Tremper. 2014. Psalms: An Introduction and Commetnary. Tyndale Old Testament Commetnaries. Downers Grove: IVP Academic.

[R38] Maccallum Fiona, and Bryant Richard A.. 2008. Self-Defining Memories in Complicated Grief. Behaviour Research and Therapy 46: 1311–15. 10.1016/j.brat.2008.09.003 18977471

[R39] Marías Julián. 1971. Metaphysical Anthropology: The Empirical Structure of Human Life. Translated by López-Morillas Frances M.. University Park: Penn State University Press.

[R40] Markovic Jelena. 2024. Transformative Grief. European Journal of Philosophy 32: 245–59.

[R41] Marušić Berislav. 2018. Do Reasons Expire? An Essay on Grief. Philosopher’s Imprint 18: 1–21.

[R42] Marušić Berislav. 2022. On the Temporality of Emotions: An Essay on Grief, Anger, and Love. Oxford: Oxford Unviersity Press.

[R43] Mathewes Charles T. 2007. A Theology of Public Life. Cambridge: Cambridge University Press.

[R44] McKirland Christa L. 2022. God’s Provision, Humanity’s Need: The Gift of Our Dependence. Grand Rapids: Baker Academic.

[R45] McNeil Michael J., Baker Justin N., Snyder Ian, Rosenberg Abby R., and Kaye Erica C.. 2021. Grief and Bereavement in Fathers After the Death of a Child: A Systematic Review. Pediatrics 147: e2020040386. 10.1542/peds.2020-040386 33648950

[R46] Middlemas Jill. 2005. The Trouble of Templeless Judah. Oxford Theological Monographs. Oxford: Oxford University Press.

[R47] Mikulincer Mario, and Shaver Phillip R.. 2022. An Attachment Perspective on Loss and Grief. Current Opinion in Psychology 45: 101283. 10.1016/j.copsyc.2021.11.003 35008028

[R48] Moller Dan. 2007. Love and Death. The Journal of Philosophy 104: 301–16.

[R49] Motto Daniel. 2024. A New Defence against the Problem of Evil. Religions 15: 1149.

[R50] Nussbaum Martha. 2001. Upheavals of Thought: The Intelligence of Emotions. Cambridge: Cambridge Unviersity Press.

[R51] O’Donovan Oliver. 2013. Self, World, and Time. Ethics as Theology. Grand Rapids: Eerdmans, vol. 1.

[R52] O’Donovan Oliver. 2017. Entering into Rest. Ethics as Theology. Grand Rapids: Eerdmans, vol. 3.

[R53] Pak Kenneth K. 2016. Divine Power and Evil: A Reply to Process Theology. London: Routledge.

[R54] Parfit Derek. 1986. Reasons and Persons. Reprint. Oxford: Oxford University Press.

[R55] Peckham John C. 2018. Theodicy of Love: Cosmic Conflict and the Problem of Evil. Grand Rapids: Baker Academic.

[R56] Radner Ephraim. 2016. A Time to Keep: Theology, Mortality, and the Shape of a Human Life. Waco: Baylor University Press.

[R57] Radner Ephraim. 2024. Mortal Goods: Reimagining Christian Political Duty. Grand Rapids: Baker Academic.

[R58] Robinaugh Donald J., and McNally Richard J.. 2013. Remembering the Past and Envisioning the Future in Bereaved Adults with and without Complicated Grief. Clinical Psychological Science 1: 290–300.

[R59] Robles Theodore F., and Kiecolt-Glaser Janice K.. 2003. The Physiology of Marriage: Pathways to Health. Physiology & Behavior 79: 409–16. 10.1016/s0031-9384(03)00160-4 12954435

[R60] Rohrbaugh Michael J., Mehl Matthias R., Shoham Varda, Reilly Elizabeth S., and Ewy Gordon A.. 2008. Prognostic Significance of Spouse We Talk in Couples Coping with Heart Failure. Journal of Consulting and Clinical Psychology 76: 781–89. 10.1037/a0013238 18837595

[R61] Rosenthal Natalie L., and Kobak Roger. 2010. Assessing Adolescents’ Attachment Hierarchies: Differences Across Developmental Periods and Associations with Individual Adaptation. Journal of Research on Adolescence 20: 678–706. 10.1111/j.1532-7795.2010.00655.x PMC3336158 22545000

[R62] Ross Allen P. 2011. A Commentary on the Psalms. Kregel Exegetical Library. Grand Rapids: Kregel Press, vol. 2 of 3.

[R63] Salters Robert B. 2011. Lamentations: A Critical and Exegetical Commentary. The International Critical Commentary. London: Bloomsbury.

[R64] Sbarra David A., and Hazan Cindy. 2008. Coregulation, Dysregulation, Self-Regulation: An Integrative Analysis and Empirical Agenda for Understanding Adult Attachment, Separation, Loss, and Recovery. Personality and Social Psychology Review 12: 141–67. 10.1177/1088868308315702 18453476

[R65] Sekowski Marcin, and Prigerson Holly G.. 2021. Associations between Interpersonal Dependency and Severity of Prolonged Grief Disorder Symptoms in Bereaved Surviving Family Members. Comprehensive Psychiatry 108: 152242. 10.1016/j.comppsych.2021.152242 33979631

[R66] Sollereder Bethany N. 2020. God, Evolution, and Animal Suffering: Theodicy Without a Fall. New York: Routledge.

[R67] Sonderegger Katherine. 2016. The Doctrine of God. Volume 1 of Systematic Theology. Minneapolis: Fortress Press.

[R68] Spaemann Robert. 2006. Persons: The Difference between “Someone” and “Something”. Translated by O’Donovan Oliver. Oxford: Oxford University Press.

[R69] Strawson Galen. 2018. Things That Bother Me: Death, Freedom, the Self, Etc. New York: New York Review of Books.

[R70] Stroebe Margaret, and Schut Henk. 1999. The Dual Process Model of Coping with Bereavement: Rationale and Description. Death Studies 23: 197–224. 10.1080/074811899201046 10848151

[R71] Stroebe Margaret, and Schut Henk. 2010. The Dual Process Model of Coping with Bereavement: A Decade On. Omega: Journal of Death & Dying 61: 273–89. 10.2190/OM.61.4.b 21058610

[R72] Stroebe Margaret, Schut Henk, and Stroebe Wolfgang. 2005. Attachment in Coping with Bereavement: A Theoretical Integration. Review of General Psychology 9: 48–66.

[R73] Stump Eleonore. 2012. Wandering in the Darkness: Narrative and the Problem of Suffering. Oxford: Oxford University Press.

[R74] Ticciati Susannah. 2022. On Signs, Christ, and the Interpretation of Scripture. Reading Augustine. London: Bloomsbury.

[R75] Trinke Shanna J., and Bartholomew Kim. 1997. Hierarchies of Attachment Relationships in Young Adulthood. Journal of Social and Personal Relationships 14: 603–25.

[R76] van der Lugt Mara. 2021. Dark Matters: Pessimism and the Problem of Suffering. Princeton: Princeton University Press.

[R77] van Inwagen Peter. 1991. The Problem of Evil, The Problem of Air, and the Problem of Silence. Philosophical Perspectives 5: 135–65.

[R78] van Inwagen Peter. 2006. The Problem of Evil. Oxford: Oxford University Press.

[R79] Vitale Vince R. 2023. Non-Identity Theodicy: A Grace-Based Response to the Problem of Evil. Oxford: Oxford University Press.

[R80] Webster John. 2012. The Domain of the Word: Scripture and Theological Reason. London: T&T Clark.

[R81] Wolterstorff Nicholas. 2019. Grief Speaks the Truth: After Loss, My Faith Endured—But It Changed. The Christian Century 136: 26–29.

